# Identification of Doxorubicin as Repurposing Inhibitory Drug for MERS-CoV PLpro

**DOI:** 10.3390/molecules27217553

**Published:** 2022-11-04

**Authors:** Ahmed L. Alaofi, Mudassar Shahid, Mohammad Raish, Mushtaq Ahmad Ansari, Rabbani Syed, Mohd Abul Kalam

**Affiliations:** 1 Department of Pharmaceutics, College of Pharmacy, King Saud University, P.O. Box 2457, Riyadh 11451, Saudi Arabia; 2 College of Pharmacy Building 23, Pharmaceutics Department, King Saud University, Ground Floor, Office AA 79, P.O. Box 2457, Riyadh 11451, Saudi Arabia; 3 Department of Phamacology and Toxicology, College of Pharmacy, King Saud University, P.O. Box 2457, Riyadh 11451, Saudi Arabia

**Keywords:** Middle East respiratory syndrome coronavirus, papain-like protease, doxorubicin

## Abstract

Middle East respiratory syndrome coronavirus (MERS-CoV), belonging to the betacoronavirus genus can cause severe respiratory illnesses, accompanied by pneumonia, multiorgan failure, and ultimately death. CoVs have the ability to transgress species barriers and spread swiftly into new host species, with human-to-human transmission causing epidemic diseases. Despite the severe public health threat of MERS-CoV, there are currently no vaccines or drugs available for its treatment. MERS-CoV papain-like protease (PLpro) is a key enzyme that plays an important role in its replication. In the present study, we evaluated the inhibitory activities of doxorubicin (DOX) against the recombinant MERS-CoV PLpro by employing protease inhibition assays. Hydrolysis of fluorogenic peptide from the Z-RLRGG-AMC–peptide bond in the presence of DOX showed an IC_50_ value of 1.67 μM at 30 min. Subsequently, we confirmed the interaction between DOX and MERS-CoV PLpro by thermal shift assay (TSA), and DOX increased Δ*T*_m_ by ~20 °C, clearly indicating a coherent interaction between the MERS-CoV PL protease and DOX. The binding site of DOX on MERS-CoV PLpro was assessed using docking techniques and molecular dynamic (MD) simulations. DOX bound to the thumb region of the catalytic domain of the MERS-CoV PLpro. MD simulation results showed flexible BL2 loops, as well as other potential residues, such as R231, R233, and G276 of MERS-CoV PLpro. Development of drug repurposing is a remarkable opportunity to quickly examine the efficacy of different aspects of treating various diseases. Protease inhibitors have been found to be effective against MERS-CoV to date, and numerous candidates are currently undergoing clinical trials to prove this. Our effort follows a in similar direction.

## 1. Introduction

Middle East respiratory syndrome (MERS), a contagious respiratory illness that often develops into acute respiratory distress syndrome (ARSD) accompanied by trauma and mostly death, was first reported in the Jeddah province in Saudi Arabia in November 2012 [1]. The disease quickly spread to other Middle Eastern countries, the Mediterranean region, Europe, the Americas, and other parts of Asia, infecting more than 8000 individuals and causing approximately 851 deaths, mostly in Middle East [2,3,4]. The recent COVID-19 pandemic has caused MERS coronavirus (MERS-CoV) to fade from public memory, but the threat of emerging MERS or its new variants is still looming. The pathogenic human coronavirus MERS-CoV killed approximately 36% of infected patients in Saudi Arabia and South Korea [5].

MERS-CoV, like other coronaviruses, has crown-like spikes on the outer surface, varying in size from 65–125 nm in diameter and containing a positive-sense single-stranded RNA (ssRNA) genome about 30 kb in size [2,6] that shares > 99% sequence identity. MERS-CoV has a low mutation rate and little variance among the genomes. During the 2012 epidemic, most humans were infected with clade B of MERS-CoV genomes, while the other clade A contains only a few strains [7]. MERS-CoV, which was transmitted from camel to human, is still prevalent in dromedary camels and can emerge at any time with drastic consequences.

The genome of MERS-CoV encodes four structural proteins: spike (S), membrane (M), envelope (E), and nucleocapsid (N) proteins, as well as 16 nonstructural proteins (nsp) in which papain-like protease (PLpro) is part of nsp3. PLpro is an essential enzyme for MERS-CoV replication and known to be an important target for MERS-CoV treatment strategies [8,9]. The structure of PLpro, a multifunctional enzyme, consists of two distinct parts: the N-terminal ubiquitin-like (Ubl) and catalytic domains. The catalytic domain compromises three subdomains: finger, thumb, and palms, while the Ubl domain consists of five β strands and two helical structures, as well as two blocking loops named BL1 and BL2. [10]. The catalytic triad Cys111, His278, and Asp293 that recognizes motif sequence (L/I)XGG↓(A/D)X is located at the center region between the thumb and palm domains [8]. The blocking loops BL1 and BL2 located in PLpro are assumed to be structurally important. For instance, PLpro inhibitors of severe acute respiratory syndrome coronavirus (SARS-CoV) bind to the BL2 loop instead of the catalytic site and thus block the entrance to the active site [8].

Currently, there is no effective antiviral drug against MERS-CoV, nor has a defensive vaccine been developed. In an attempt to combat this dreadful disease, a continuous robust mechanism to develop therapeutics is needed to overcome the unseen adversity. Among the best-known and practical pharmacological targets in antiviral therapy are the proteases of the human immunodeficiency virus [11], hepatitis C viruses [12,13], dengue virus and influenza virus [14,15]. Coronaviral proteases such as 3-chymotrypsin-like protease (3CLpro) and papain-like protease (PLpro) are considered to be effective antiviral targets since they are large precursor proteins that are cleaved to generate mature nonstructural polyproteins (pp1a and pp1ab) [16,17]. PLpro are primary targets for nidoviruses, specifically coronavirus. Shen et al. showed activity of the drug GRL0617 against SARS-CoV-2 PLpro [18]. In silico and in vitro reports have shown the activity of many drugs, such as the hypertension drug tanshinone and its derivatives [19,20,21], hepatitis C virus (HCV) drugs asunaprevir, simeprevir, and grazoprevir [22], and famotidine, an FDA-approved drug to treat heartburn and ulcers [23], against SARS PLpro and can be repurposed for the administration of coronavirus pathogenesis. Similarly, the anticancer drug disulfiram has been identified to inhibit PLpro of SARS-CoV, SARS-CoV-2, and MERS-CoV [21,24].

Since there is no vaccine or specific treatment for MERS-CoV, it is essential to identify therapeutic options to both limit the replication of this virus and prevent its spread. Based on the extensive homology between severe acute respiratory syndrome coronavirus (SARS-CoV), MERS, and SARS-CoV-2, we hypothesize that specific targeted drugs can be repurposed to inhibit replication and prevent the spread of these viruses. Therefore, doxorubicin (molecular formula: C_27_H_29_NO_11_), which is commonly used to treat several types of cancer, was selected to check its ability to inhibit MERS-CoV PLpro. For the majority of malignancies, doxorubicin should be administered intravenously at doses between 40 and 75 mg/m^2^ [25].

Doxorubicin has the capacity to produce free radicals that lead to DNA and cell membrane damage [26,27]. In addition to causing free radical-mediated oxidative damage to DNA, doxorubicin also induces cardiotoxicity and nephrotoxicity [28]. In recent high-throughput screening (HTS), DOX was shown to have promising antiviral activity against SARS-CoV-2 in Caco-2 cells and HEK293 cells [29]. In another in silico study, doxorubicin was found to interact with the main protease and inhibit SARS-CoV-2 replication, but no study has been conducted on MERS-CoV [29,30,31].

## 2. Results

### 2.1. Expression and Purification of MERS-CoV PLpro

**Figure 1:**
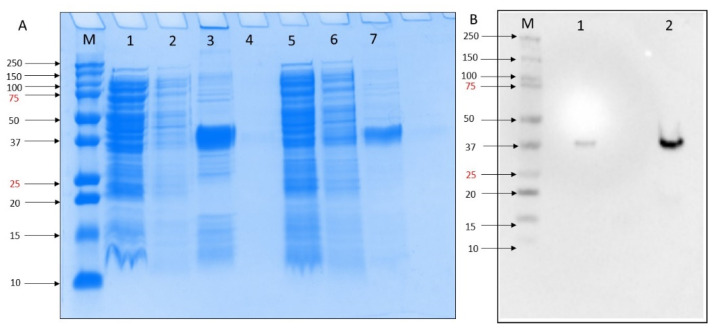
(**A**) SDS-PAGE 10% analysis of small-scale expression for the MERS-PLpro with poly His-tag of E. coli BL21(DE3) molecular weights for MERS-PLpro: 37. kDa. M = Precision Plus Protein^TM^ all blue standard protein marker from BioRad, lane 1 = crude extract, lane 2 = flowthrough, lane 3 = elution after 10 mM imidazole wash, lane 4 = blank, lane 5 = crude extract, lane 6 = flowthrough, lane 7 elution after 30 mM imidazole wash. (**B**). Western blot M = Precision Plus Protein^TM^ all blue standard protein marker from BioRad, lane 1 = crude extract, lane 2 = elution after 30 mM imidazole wash.

To test the inhibitory activity of DOX against the MERS-CoV, we first expressed PL protease in *E. coli*. Expression was confirmed by SDS-PAGE analysis in which a 37 kDa recombinant protein was produced by growing in Magic Media at 30 °C 18 h. The purification column process resulted in more purified rMERS-PLpro protein (Figure 1A, lane 7) compared to washing buffer (Figure 1A, lane 3). We obtained a high-level MERS-PLpro gene expression of in BL21 DE3 *E. coli* self-induced culture. Western blot analysis of the MERS-PLpro with N-terminal His-Tag was performed using His-tag primary monoclonal antibody (SC-8036) and confirmed the presence of MERS-PLpro recombinant protein (Figure 1B).

### 2.2. MERS-CoV PLpro Inhibition Assay

**Figure 2:**
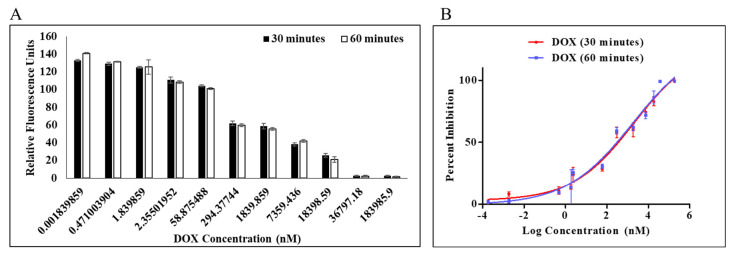
Percentage inhibition of MERS-CoV PLpro activity as a function of doxorubicin log concentration (**A**). Percentage inhibition for dose–response curve (**B**). Dose–response curve of MERS-CoV PLpro inhibition identified from the IC_50_ protease inhibition assay with dissociation of Z-RLRGG-AMC substrate. Values represent the average ± standard deviation of three replicates.

A PLpro inhibition assay was performed to determine the efficacy of doxorubicin as an inhibitor against PLpro protease. Dose-dependent inhibition of PLpro by DOX was measured by protease inhibition assay in which fluorescence from AMC was continuously monitored. Enzymatic activity was measured using a previously reported procedure for the continuous enzymatic inhibition experiment [17,32], and by holding the amounts of the enzyme and substrate constant while changing the quantity of chemical, inhibition curves were generated (Figure 2). Enzymatic inhibition activity of DOX at concentration 1.84 pM–183.98 µM was calculated at the initial slope in each curve (Figure 2B). It is interesting to note that DOX had a dose-dependent impact on the activity of the PLpro enzyme (Figure 2A). The estimation of an apparent inhibition at various dose and time points was made by the nonlinear regression analysis using a straightforward inhibition model. This apparent inhibition was noted in the IC_50_ values of 1.67 and 1.79 μM at 30 min and 60 min, respectively. Both 30 and 60 min were reported to evaluate the effect of incubation time on the protease inhibition. DOX can be expected to bind to the active site and behave as a competitive inhibitor (Table S1). The change in IC_50_ post 30 min of incubation was insignificant from 60 min.

### 2.3. Thermal Shift Assay (TSA)

**Figure 3:**
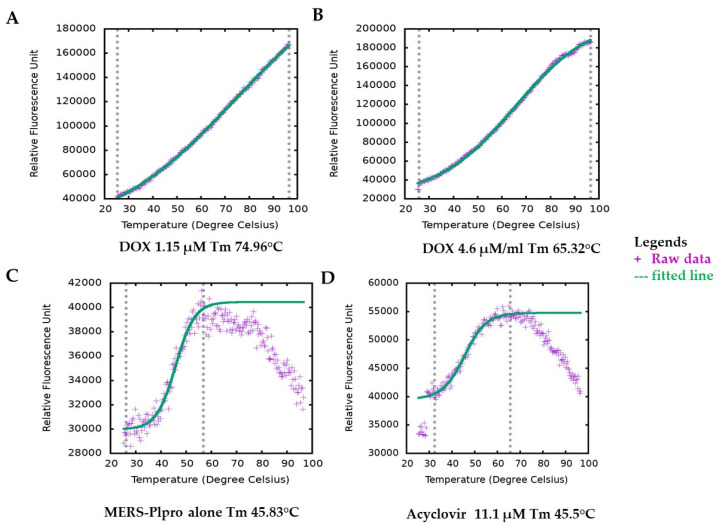
Melting temperature (Tm) of MERS-CoV-2 PLpro by thermal shift assay (TSA). Figure plotted by TSA-CRAFT. Raw data represented with plus sign (light blue) and fitted data with dashed line (light red). Representative images of Tm of MERS-CoV PLpro profile with DOX at different concentrations (**A**,**B**). The melting temperature of MERS-CoV-2 PLpro only (**C**) and with acyclovir showed similar melting temperatures (**D**).

DOX has no intrinsic fluorescence or quenching effects that overlaps the fluorophore emission, which can interfere with the inhibition assay signals. Still, we followed our inhibition assay finding with a secondary assay with different fluorescence emission spectrum and detection method thermal shift assay (TSA), where we observed undifferentiated results. Generally, the interaction of active molecules with a protein resulted in a change in the stability of the protein against physical, thermal or chemical denaturation. The thermal shift assay (TSA), also known as differential scanning fluorimetry (DSF), uses fluorescence to monitor changes in protein stability brought on by ligands or active molecules [33]. By monitoring the protein’s apparent melting temperature (Tm) in the presence of increasing concentrations of an interacting molecule, it is possible to evaluate the protein’s thermal stability using a fluorescence probe such as SYPRO orange that tracks the development of the protein unfolding process. DOX, in a concentration-dependent manner, altered the thermal stability of MERS-CoV PLpro, as evident from the results (Figure 3). Ligand-induced destabilizing effects on a protein are not unusual [34,35,36]. The variation in melting temperature (ΔTm) (mean = 21.058 °C) for various concentrations for DOX ranged from 16.26 °C to 23.4 °C. Their enzymatic protease inhibition is compatible with the thermal shift assay. In contrast, DOX enhanced protein stability by about 20 °C in the TSA experiment and exhibited an IC_50_ value of 1.67 and 1.79 μM (Table S1 and Figure 2) in protease inhibition. Interestingly, both of our tests indicating the effectiveness of DOX in suppressing MERS-CoV-2 PLpro are consistent with it being dose-dependent. It is worth mentioning that acyclovir (antiviral agent) did not change at any concentration the Tm of MERS-CoV PLpro; indicating the possibility of no interaction between acyclovir and PLpro (Figure 2D).

### 2.4. Doxorubicin Binding Site

**Figure 4:**
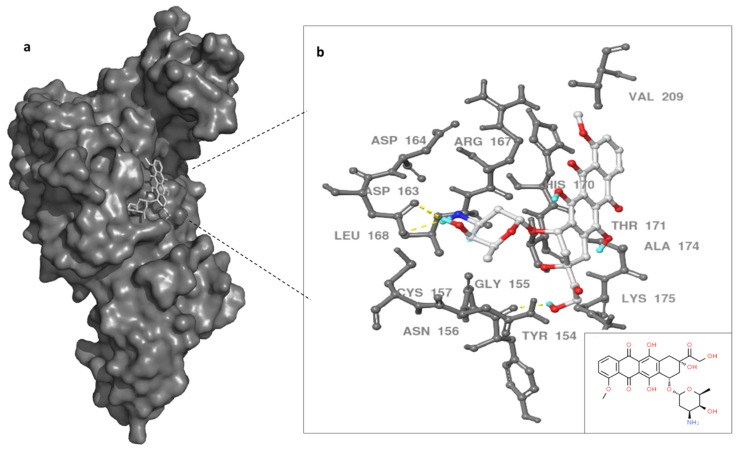
Binding site of doxorubicin (white) with MERS-CoV PLpro surface representation (dark gray) (**a**). In (**b**), enlargement of the PLpro–doxorubicin interaction and PLpro residues are noted in gray and dashed yellow line indicate hydrogen bonds. Doxorubicin in white, small box on the left for 2D doxorubicin. Three hydrogen bonds formed between DOX and PLpro indicated with yellow dashed line.

The binding site of DOX on MERS-CoV PLpro was evaluated utilizing MD simulations data and docking techniques. Autodock vina and SwissDock showed similar binding site for DOX on the protease target. Previous work showed SARS inhibitors bind to BL2 instead of catalytic triad in a slightly similar region of our docking results [37]. Therefore, we proposed the binding site of DOX was in the thumb domain of MERS-CoV PLpro (Figure 4). MERS-CoV PLpro Y154, and D163, residues formed hydrogen bonds with primary amine (groove binding) and anchor of doxorubicin molecule, while G155, R167, V209, K175, and A174 residues provided the hydrophobic interactions with doxorubicin (Figure 4). Although other possible binding sites of doxorubicin can be proposed, the thumb domain might be the most probable binding site for DOX. This is due to the thumb domain being nearest to the catalytic site of the MERS-CoV PLpro, as well as doxorubicin being able to induce the stability of MERS-CoV PLpro, as our thermal assay results suggest. Moreover, the docking results showed a similar binding site using the X-ray structure (data not shown) of PLpro as the 100 ns dynamic structure of PLpro.

### 2.5. MERS-CoV PLpro Flexibility

**Figure 5:**
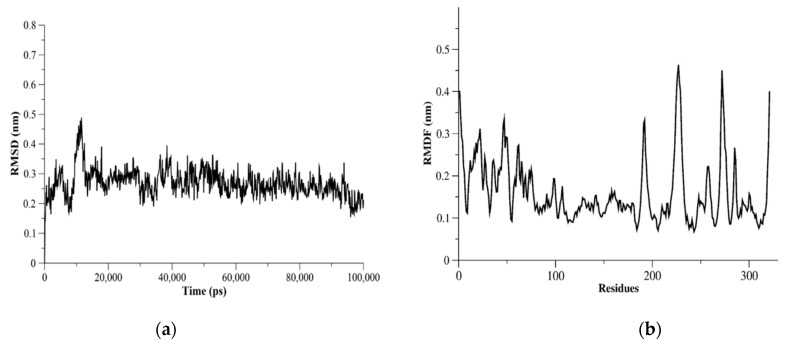
MERS-CoV PLpro MD simulation results. In (**a**), C-α root-mean-square deviation (C-α RMSD) as a function of time (ps) is plotted. C-α RMSD showed a stable system during the 100 ns-run simulations. (**b**) The C-α root-mean-square fluctuation (C-α RMSF) plotted against PLpro residues showed flexible residues and regions.

The dynamics of proteases might be helpful in repurposing or designing new antiviral agents as well as improving the accuracy of binding site identification. Therefore, we assessed the flexibility of structurally important loops and domains of MERS-CoV PLpro using MD simulations. Measurements of root-mean-square fluctuation (RMSF) for MERS-CoV PLpro showed flexible residues were K22, N23, E46, K47, D98, K99, K138-D143, N191, V192, Y223-E230, Ile271-Val275, and Asp319-Asn321 (Figure 5). The I271-V275 residues were in the BL2 loop and could be seen as a flexible region with RMSF > 3.0 Å. The catalytic triad residues (Cys110, His277, Asp292) did not show flexible residues based on the RMSF measurements for MERS-CoV PLpro monomer. The RMSF results of L105, D146, P162, P249, and G276 residues showed these residues were less flexible (RMSF < 3.0 Å).

## 3. Material and Method

### 3.1. Chemicals

Doxorubicin hydrochloride was purchased from Biosynth, Carbosynth Ltd. (Compton, UK); Z-Arg-Leu-Arg-Gly-Gly-AMC acetate was purchase from Bachem (Torrance, CA, USA). MagicMedia™ E. coli expression medium, SYPROTM orange protein gel stain and Pierce™ BCA protein assay kit were purchased from Invitrogen by Thermo Fisher Scientific (Rockford, IL, USA). Affinity column HisGraviTrap Cytiva was obtained from (Uppsala, Sweden). Precision Plus ProteinTM all blue standard protein marker, Every Blot Blocking Buffer and nitrocellulose membrane from BioRad (Hercules, CA, USA) and sample buffer, Laemmli 2X, Benzonase® Nuclease from Sigma (Marlborough, MA, USA), Antibodies against primary monoclonal-His-tag and horseradish peroxidase-conjugated secondary antibody were purchased from Santa Cruz Biotechnology (Santa Cruz, CA, USA), and the rest of the chemicals used in this study were obtained from standard commercial suppliers.

### 3.2. Protease Expression Plasmids

Codon-optimized and synthesized by GenScript, the MERS-CoV PLpro sequence (GenBank accession number AFS88944) was then cloned into pET28b+ with NcoI and XhoI carrying a C-terminal His-tag.

### 3.3. SDS-PAGE

SDS polyacrylamide gel electrophoresis using BioRad Mini-protean: protein samples were mixed with a buffer containing 2X Laemmlli Buffer (Sigma, Marlborough, MA, USA) in a total volume of 20 μL. Samples were heated at 95 °C in a water bath for five minutes and immediately cooled in ice before electrophoresis on SDS-PAGE initially at 60 V, and after the protein passed the stacking gel it was run at 100 V. After electrophoreses, gels were stained with Coomassie blue staining solution and destained in a gentle agitation with a destaining solution of 50% H_2_O, 10% acetic acid and 40% methanol. Pictures of SDS-PAGE gels were taken and cropped using the Gel-Doc System (BioRad, Hercules, CA, USA) and made publication-ready in Microsoft PowerPoint without any color or contrast correction.

### 3.4. Western Blotting

Western blotting was performed for monoclonal-His antibody to confirm the presence of N-terminal 6xHis-Tag, as described previously [38,39]. Whole cell protein (10 μL) isolated by sonication was mixed with 2X Laemmli dye and electrophoresed on 10% SDS−polyacrylamide gel, after which it was transferred onto nitrocellulose membranes using a transblot turbo machine (BioRad), then blocked in Every Blot Blocking Buffer at room temperature for 10 min (BioRad). The membranes were washed five times with TBST buffer at 5 min intervals, then incubated overnight (4 °C) with primary poly-His antibody. After 5 consecutive washes at 5-minute intervals with TBST, the membranes were then rinsed with TBST 5 times for 5 min each after being incubated with secondary antibody for 2 hours at room temperature. Bands were scanned using Luminata™ Western Chemiluminescent horse radish peroxidase substrates (Millipore, Billerica, MA, USA), followed by Gel-Doc System (BioRad, Hercules, CA, USA).

### 3.5. Protein Purification

Plasmid-transformed BL21(DE3) *Escherichia coli* cells were cultured in Magic Media (Invitrogen, Waltham, MA, USA) at 30 °C for 18 h with kanamycin 50 µg/mL. At 6000 RPM and 4 °C, the media containing cells were centrifuged to form a pellet and kept at −80 °C till further use. The cell pellet was reconstituted in lysis buffer (50 mM Tris-HCl, 250 mM NaCl, 10 mM imidazole, 2 mM beta-mercaptoethanol, 5% (*v*/*v*) glycerol, phenylmethylsulfonyl fluoride, pH 8.0) and then lysed by ultrasonically processing it three times for five seconds each at a 30% amplitude (Ultrasonic Processor, Gex 130, USA) and centrifuged at 25,000 RPM for 30 min. The 5 mL HisGraviTrap (GE Healthcare, Chicago, IL, USA) was used to purify His-tag proteins, and before loading the cell lysate, the column was equilibrated with 5CV of lysis solution. The tagged proteins were eluted with a lysis buffer containing 200 mM imidazole after the column had been rinsed with the same lysis buffer with 30 mM imidazole without PMSF. The cell free extract and elute were collected for analysis by SDS-PAGE. The elutions were further dialyzed overnight with 14,000 Dalton MWCO dialysis tubing (D9527, Sigma) containing 20 mM Tris-buffer, pH 8.0. For every inhibition and binding assay, fresh protein was eluted and concentration estimated with Pierce™ BCA Protein Assay Kit Thermo Fisher scientific.

### 3.6. MERS-CoV PLpro Inhibition Assay

IC_50_ values for different concentrations and durations of DOX were determined using the plate-based flat bottom black test 384. Briefly, hydrolyzed fluorogenic peptide Z-Arg-Leu-Arg-Gly-Gly peptide linkage 7-amido–methylcoumarin (Z-RLRGG-AMC) significantly increased the fluorescence of the AMC fraction, allowing the conversion to be measured accurately. Reactions were performed in a total volume of 50 µL, which contained the following components: 20 mM Tris buffer, pH 8.0, 30 µM Z-RLRGG-AMC, and varying concentrations of inhibitor (DOX) (1.84 pM–183.98 µM). Assays were initiated with the addition of PLpro to produce a final enzyme concentration of 60 nM. Plates were shaken gently for 30 s, and fluorescence from the release of AMC from peptide recorded for 60 min on Biotek HT Microplate Reader (USA) (*γ*_excitation_  =  360 nm; *γ*_emission_  =  460 nm and gain  =  40) and the IC_50_ value was calculated by the dose–response-inhibition function in Graphpad Prism with inhibitor vs. normalized response equation. The experiment was repeated three times. Positive control wells, representing 100% inhibition, included 10 μg/mL DOX; negative control wells, showing zero inhibition, included vehicle contained only buffer.

### 3.7. Thermal Shift Assay (TSA)

Doxorubicin binding to MERS CoV PLpro protein was estimated by differential scanning fluorimetry (DSF) using an Applied Biosystems™ PCR machine as previously described [40]. We also tested acyclovir’s (known antiviral agent) possible interaction against MERS-CoV PLpro as DOX. Briefly, reaction buffer was used as a control and MERS CoV-PLpro protein was diluted into it to a final concentration of 2 mol/L before being treated at 25 °C for 30 min with 0.29–18.4 μM of DOX. A temperature gradient of 1 °C/minute between 25 °C and 95 °C was used to record the fluorescence using SYPRO orange (2, Thermal Fisher, S6650). The melting temperature (*T*_m_) was determined with TSA CRAFT software, which is robust and less sensitive to noise (Lee et al., 2019). TSA-CRAFT, implement the curve fitting through the Boltzmann equation to determine the melting temperature (*T*_m_):

FDinv(*Tn*) = −f(*Tn* + 1) − f(*Tn* − 1)2Δ*T* (1)

f(T) = fmin + fmax − fmin1 + exp*T*_m_ − *Ta* (2)

### 3.8. Molecular Dynamic (MD) Simulations

Molecular structure simulated in a box of water might reveal better 3D structure in comparison to crystal (X-ray) structure of large molecules, such as proteins and proteases. Here, the X-ray structure for MERS-CoV PLpro PDB file was obtained from Protein Data Bank (ID: 4RNA) and simulated in a box of water. Following our previous work [41,42,43,44], the PDB file was used as a starting structure for molecular dynamic (MD) simulations for PLpro in a cubic box of TIP3P water. The MERS-CoV PLpro was solvated with 29,116 TIP3P water in a size of 9.77827 nm. The ionic strength simulations were carried out with 0.15 M. A 84 Na^+^ and 84 Cl^−^ ions were added by replacing water molecules. The MERS-CoV PLpro simulation was conducted at pH 7.0 and titratable residues (Glu, Asp, His, Lys and Arg) were assigned corresponding to pH 7.0 condition. MD simulations of MERS-CoV PLpro for 100 ns were conducted at a constant temperature of 300 K. GROMACS 5.1.4 [45,46] with CHARMM35 force filed and unbonded interaction were used for the MD simulations. The dynamic structure (frame) produced from MD simulations of MERS-CoV PLpro at 100 ns was obtained for binding site analysis.

### 3.9. Docking of Doxorubicin to MERS-CoV PLpro

The obtained dynamic MERS-CoV PLpro structure at 100 ns was used for docking using Autodock vina and SwissDock programs [47]. The 100 ns structure of MERS-CoV PLpro and the ligand doxorubicin structure [48] were used to identify the DOX binding sites. Docking was performed following the Autock vina manual [49]. Briefly, polar hydrogens charges were added using Kollman Charges. The grid box was in the center of MERS-CoV PLpro of X −8.578, Y 17.895, and Z 8.150 Å and spacing of 0.375 Å. The 100 ns MERS-CoV PLpro (target) and DOX (ligand) were uploaded to the SwissDock webserver. The selected PLpro-DOX binding sites were proposed based on the pose similarity of the binding sites obtained from both techniques and not only on highest scores. Finally, the docking results were visualized and analyzed using PyMOL and Maestro Schrödingerprogram [50,51].

## 4. Discussion

The mayhem caused by the COVID-19 pandemic has amplified the need for developing pharmacologically active drugs against MERS-CoV. The repository of active compounds needs to be active via different mechanism with high efficacy and minimal susceptibility to drug resistance and viral mutations. Drugs acting against coronavirus proteases address some of the problems, as these regions are highly conserved in various strains. Repurposing or development of drugs can be started with small- or large-scale screening from experimental and/or computational methods. Similar pipelines have been successfully developed in the past [52,53,54,55,56,57,58]. Here, we have applied a similar approach to screen the ability of DOX against MERS-CoV PL protease.

A peptidic FRET substrate called Z-RLRGG-AMC, a continuous enzyme assay, was employed in the screening test for DOX against MERS-CoV PLpro to determine how much DOX interfered with the enzyme’s ability to function. After DOX had been successfully screened for its dose-dependent ability to inhibit MERS-CoV PLpro, the protein’s thermal stability against thermal denaturation was determined (using fluorescence TSA). Although TSA is a quick and reliable method, its limitations include the frequent occurrence of false-negative results and the fact that binding affinity does not always transfer into an inhibitory effect [59]. On the other hand, methods based on protein activity detection offer clear evidence of inhibition during screening, but measuring the protein target’s activity requires a quick and appropriate method, which is not always straightforward. DOX demonstrated continuous inhibition at varied concentrations (1.84 pM–183.98 µM), and the thermal shift experiment revealed that the Tm ranged from 16.26 °C to 23.4 °C (TSA). The binding affinity clearly showed that DOX is a good candidate for future optimization and preclinical investigations, and the apparent change in the unfolding temperature of MERS-CoV PLpro may be caused by the specific and understandable interaction of DOX with the protease.

The sum of a ligand’s stabilizing affect is decided by the exact set of conformational states that connect specifically with the ligand over the complete conformational scene of the protein. The impact on protein steadiness will be negligible in the event that the ligand is interatomic with most conformational states with comparable authoritative affinities. In the event that the ligand is interatomic with mostly unfurled shapes, as well as the native state, a destabilizing affect might be anticipated in the event that the partiality for the nonnative states is bigger. It is possible for PLpro thumb domain to be the binding site for the doxorubicin molecule to inhibit the protease catalytic activity. Binding to this region might also stabilize the tertiary structure (i.e., increase the Tm) of MERS-CoV PLpro, as our thermal stability assay indicated. The interaction of DOX with MERS-CoV PLpro was monitored to accumulate an additional piece of evidence for their interaction. Our flexibility assessment of MERS-CoV PLpro revealed a dynamic nature for PLpro; therefore, doxorubicin interaction with thumb domain residues might hinder the entrance to the catalytic site of PLpro. This might be attributed to the interaction of doxorubicin with D163 (via hydrogen bond) next to D164 residue, which is important for MERS-CoV PLpro catalysis, along with P162, L105, P249, and G276 residues. Lin et al. showed two mutations of P162 and L105 significantly effecting PLpro catalytic activity [60].

Given that DOX is an anticancer drug with well-known pharmacokinetic and pharmacodynamic properties, it is possible to repurpose it for the treatment of MERS-CoV. It is important to note that experimental evidence corroborating our binding tests for the inhibitory effects of derivatives of DOX against dengue and yellow fever viruses has already been documented [61]. The information presented here provides verifiable experimental proof of DOX’s ability to target MERS-CoV PLpro and inhibit its catalytic activity.

## 5. Conclusions

Papain-like proteases are arguably the most widely exploited pharmacological targets to counter coronaviruses. PLpro inhibitors have been discovered using a variety of drug-discovery approaches. To speed up the drug-development process, researchers from all around the world are racing to share their findings with the scientific community. We tested doxorubicin against the purified MERS-CoV PLpro to harbor its enzymatic activity. The inhibitory activity of doxorubicin can potentially be investigated in the future in clinical trials to treat infected patients with MERS-CoV. Still, in vitro studies, findings, and further investigation by cell-based and in vivo assays are required to determine the exact dose for human administration against MERS-CoV PLpro. However, our reported IC_50_ (1.67 µM) for DOX suggested the feasibility of using DOX as an antiviral compound like the GRL0617 inhibitor (IC_50_ 2.1 µM) of SARS-CoV-2 PLpro. The proposed binding site of doxorubicin can be explained by DOX-induced stability for MERS-CoV PLpro. Both target selectivity and cellular target engagement should be considered during pharmacological characterization. Overall, we expect that our research will raise scientific knowledge of the promiscuous MERS-CoV-2 PLpro inhibitors and encourage more medication development.

## Data Availability

Data are available within the article or Supplementary Materials.

## Supplementary Materials

The following supporting information can be downloaded at https://www.mdpi.com/article/10.3390/molecules27217553/s1. Table S1: IC50 of MERS-CoV PLpro inhibition identified by dissociation of Z-RLRGG-AMC substrate against DOX at different time intervals.
