# Supplementary material for: Identification of Doxorubicin as Repurposing Inhibitory Drug for MERS-CoV PLpro
Source: Molecules. 2022 Nov 4;27(21):7553. doi: 10.3390/molecules27217553 (PMC9654812; doi:10.3390/molecules27217553)
Supplement: Supplementary file 1 [file molecules-27-07553-s001.zip › molecules-1961735-supplementary.pdf]

molecules

MDPI

Article

Identification of Doxorubicin as Repurposing Inhibitory Drug for MERS-CoV PLpro

Ahmed L. Alaofi [1,*], Mudassar Shahid [1], Mohammad Raish [1], Mushtaq Ahmad Ansari [2], Rabbani Syed [1] and Mohd Abul Kalam [1]

1. Department of Pharmaceutics, College of Pharmacy, King Saud University, P.O. Box 2457, Riyadh 11451, Saudi Arabia
2. Department of Phamacology and Toxicology, College of Pharmacy, King Saud University, P.O. Box 2457, Riyadh 11451, Saudi Arabia

* Correspondence: ahmedofi@ksu.edu.sa; King Saud University, Riyadh KSA, Pharmaceutics Department, College of Pharmacy Building 23, Ground Floor, Office AA 67. P.O. Box 2457 Riyadh 11451, Saudi Arabia (Tel) +966114677364 (Fax) +966114676383

Citation: Alaofi, A.L.; Shahid, M.; Raish, M.; Ansari, M.A.; Syed, R.; Kalam, M.A. Identification of Doxorubicin as Repurposing Inhibitory Drug for MERS-CoV PLpro. *Molecules* **2022**, *27*, 7553. https://doi.org/10.3390/molecules27217553

Academic Editors: Rosanna Di Paola and Rosalba Siracusa

Received: 24 September 2022
Accepted: 30 October 2022
Published: 4 November 2022

Publisher's Note: MDPI stays neutral with regard to jurisdictional claims in published maps and institutional affiliations.

Copyright: © 2022 by the authors. Licensee MDPI, Basel, Switzerland. This article is an open access article distributed under the terms and conditions of the Creative Commons Attribution (CC BY) license (https://creativecommons.org/licenses/by/4.0/).

Abstract: The Middle East Respiratory Syndrome coronavirus (MERS-CoV), belonging to betacoronavirus genus can cause severe respiratory illnesses, accompanied by pneumonia, multi-organ failure, and ultimately death. Coronaviruses (CoVs) have the ability to transgress-species barrier and spread swiftly into new host species especially human to human transmission causing epidemic diseases. Despite the severe public health threat of MERS-CoV there are currently no vaccines or drugs available for its treatment. MERS-CoV papain-like protease (PLpro) is a key enzyme that plays an important role in its replication. In the present study, we evaluated the inhibitory activities of Doxorubicin (DOX) against the recombinant MERS-CoV PLpro by employing protease inhibition assay, the hydrolysis of fluorogeneic peptide from Z-RLRGG-AMC–peptide bond in presence of DOX showed IC_{50} value of 1.67 μM at 30 minutes. Subsequently, we confirmed the interaction between DOX and MERS-CoV PLPro by Thermal shift assay (TSA), DOX increased ΔT_m by ~20ºC clearly indicating a coherent interaction between the MERS-CoV PL protease and DOX. The binding site of DOX on MERS-CoV PLpro was assessed using docking techniques as well as molecular dynamic (MD) simulations. DOX bind to the thumb region of the catalytic domain of the MERS-CoV PLpro. Moreover, MD simulations results showed flexible BL2 loops as well as other potential residues such as R231, R233, and G276 of MERS-CoV PLpro. Development of drug repurposing is a remarkable opportunity to quickly examine the efficacy of different aspects of treating various diseases. Protease inhibitors have been found to be effective against MERS-CoV to date, and numerous candidates are currently undergoing clinical trials to prove this and our effort is in similar direction.

Keywords: Middle East Respiratory Syndrome coronavirus; Papain-like protease; Doxorubicin.

Table S1. IC_{50} of MERS-CoV PLpro inhibition identified by dissociation of Z-RLRGG-AMC substrate against DOX **at different time intervals.**

	**30 minutes**	**60 minutes**
**IC_{50} μg/mL**	1.992	1.705
**R^2**	0.8857	0.8619
